# Reactive Oxygen Species as the Brainbox in Malaria Treatment

**DOI:** 10.3390/antiox10121872

**Published:** 2021-11-24

**Authors:** Chinedu Ogbonnia Egwu, Jean-Michel Augereau, Karine Reybier, Françoise Benoit-Vical

**Affiliations:** 1 PharmaDev, UMR 152, Université de Toulouse, IRD, UPS, 31400 Toulouse, France; chinedu.egwu@funai.edu.ng; 2 Medical Biochemistry, College of Medicine, Alex-Ekwueme Federal University, Ndufu-Alike Ikwo, P.M.B. 1010, Abakaliki, Nigeria; 3 CNRS, LCC, Laboratoire de Chimie de Coordination, Université de Toulouse, 31077 Toulouse, France; jean-michel.augereau@lcc-toulouse.fr; 4 Institut de Pharmacologie et de Biologie Structurale, IPBS, Université de Toulouse, CNRS, UPS, 31077 Toulouse, France; 5 MAAP, New Antimalarial Molecules and Pharmacological Approaches, ERL 1289 Inserm, 31077 Toulouse, France

**Keywords:** ROS, antimalarials, *Plasmodium falciparum*, malaria and oxidative stress

## Abstract

Several measures are in place to combat the worldwide spread of malaria, especially in regions of high endemicity. In part, most common antimalarials, such as quinolines and artemisinin and its derivatives, deploy an ROS-mediated approach to kill malaria parasites. Although some antimalarials may share similar targets and mechanisms of action, varying levels of reactive oxygen species (ROS) generation may account for their varying pharmacological activities. Regardless of the numerous approaches employed currently and in development to treat malaria, concerningly, there has been increasing development of resistance by *Plasmodium falciparum*, which can be connected to the ability of the parasites to manage the oxidative stress from ROS produced under steady or treatment states. ROS generation has remained the mainstay in enforcing the antiparasitic activity of most conventional antimalarials. However, a combination of conventional drugs with ROS-generating ability and newer drugs that exploit vital metabolic pathways, such antioxidant machinery, could be the way forward in effective malaria control.

## 1. Introduction

Malaria is a vector-transmitted parasite disease that continues to plague mankind. It is caused in humans by five main species of *Plasmodium.* The World Health Organization estimated a global prevalence of 229 million cases in 2019, with Sub-Saharan Africa taking the top spot, with more than 90% of the global burden and most of the deaths being due to the parasite *Plasmodium falciparum*. *Plasmodium vivax* is also a notable species and, although it is less deadly, it still has a very significant economic impact [1]. The physiopathology of *P. falciparum* and *P. vivax* malaria relies to a large extent on the oxidative stress generated by the parasites during their erythrocytic cycle [2,3,4].

It is worrisome to note that the control of malaria has stalled since 2014 [1,5], which calls for a doubling of efforts by all stakeholders. Several recommendations have been made with regard to malaria control. These recommendations range from the use of insecticide spray and sleeping under insecticide-treated nets to the use of chemotherapeutic agents. 

Regardless of these numerous interventions, the seeming lack of progress may be attributed to many factors, which may include, but are not limited to, poverty, poor sanitation, and inadequate or nonoperational health policies [6,7]. Moreover, overuse, inadequate or incomplete treatment regimens, and counterfeit drugs, which lead to drug failures and the development of resistance by both mosquitoes and malaria parasites to insecticides and antimalarials, respectively, are also responsible for this scenario [1,8,9]. Chloroquine and most antiplasmodial drugs have lost their usefulness in malaria control due to parasite resistance development [10,11], and it is worrisome to note that the continued relevance of the current gold-standard drug artemisinin and its derivatives is threatened by resistance development, which was first reported in 2008 [12,13]. Resistance of the parasite to antimalarials could be a result of its ability to develop diverse mechanisms to evade death, which can be preventive, reductive, or reparative. 

As for any cell, the maintenance of redox homeostasis is vital for parasites. The overall level of ROS in a biological system is determined by its level of production and/or elimination by the antioxidant machinery of the cells [14]. The ROS generated are very reactive and oxidize any biomolecules around their site of production [15], leading to intense cellular damage.

Since the majority of conventional antimalarials kill parasites via direct or indirect overproduction of reactive oxygen species (ROS) [16], the present article seeks to review the generation and modulation of ROS in steady state and during treatment with different antimalarials, especially artemisinin and its derivatives in relation to *P. falciparum* resistance to these drugs. An in silico comparison of the enzymes of the *P. falciparum* redox system with the *P. vivax* proteins has shown a certain level of homology between these two parasite species [17]. However, as the in vitro culture of *P. vivax* has not yet been mastered, the redox system of *Plasmodium* has almost exclusively been studied in *P. falciparum.*

## 2. Defining ROS in Living Cells

**Figure 1:**
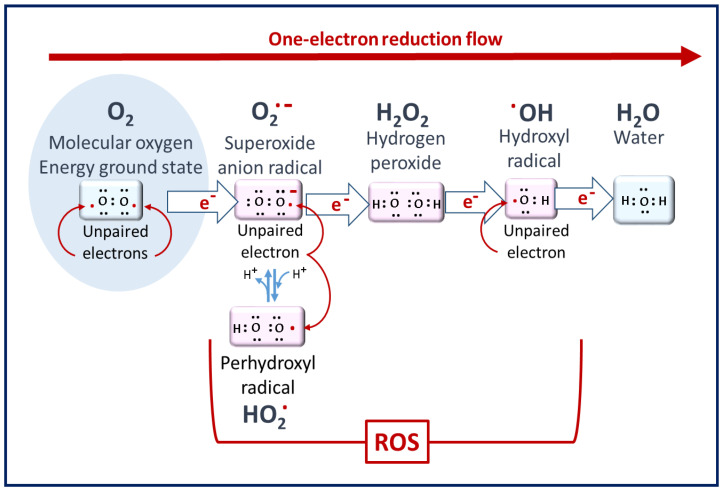
Molecular oxygen to ROS. Superoxide radicals are the primary ROS but have poor reactivity. Hydrogen peroxide is the least reactive ROS. At the end of the reduction flow, hydroxyl radicals are the most reactive [14].

ROS are defined as oxygen-containing reactive species. This term includes superoxide radicals (O_2_^•−^), hydrogen peroxide (H_2_O_2_), hydroxyl radicals (^•^OH), singlet oxygen (^1^O_2_), peroxyl radicals (LOO^•^), alkoxyl radicals (LO^•^), lipid hydroperoxide (LOOH), and peroxynitrite (ONOO^−^), among others [15]. Their generation can first occur as a result of the partial reduction of oxygen, as described in Figure 1. Among the ROS, some species are radicals, i.e., have unpaired electrons in their outer orbit—for example, superoxide and hydroxyl radicals. The different forms of ROS have varying levels of reactivity depending on their oxidation potential, with H_2_O_2_ being the least reactive and ^▪^OH being the most reactive.

## 3. The Biochemical Impacts of ROS in Living Cells: Is There Any Peculiarity in Malaria?

**Figure 2:**
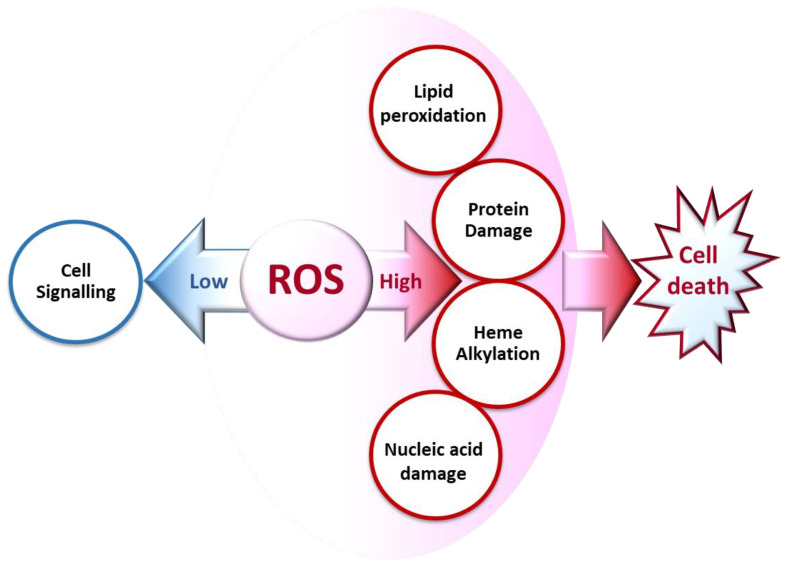
Biochemical impacts of ROS depending on their concentrations.

ROS generated at low concentrations under physiological conditions are often beneficial, playing important roles as regulatory mediators in signaling processes [15,18]. Nevertheless, at high concentrations, they become deleterious for the cells. The toxicity of free radicals stems from their unstable nature and predisposition to donate or abstract electrons from nearby molecules, triggering a chain reaction that can be terminated by another molecule with unpaired electrons. Lipids, proteins, and nucleic acids are attacked and damaged by ROS, which affects the survival of living organisms [14,15] (Figure 2).

This overproduction of ROS can be endogenous due to the dysregulation associated with pathological processes such as aging, inflammation [19,20], or cardiovascular diseases [21] and can also be exogenous with the administration of xenobiotics such as antimalarials [22].

### 3.1. Cell Signaling

ROS play important physiological roles, such as in the induction of apoptosis and suppression of some genes’ expression, at low concentrations [18]. Studies have reported the production of ROS by specialized plasma membrane oxidases and nicotinamide adenine dinucleotide phosphate (NADPH) oxidases in normal physiological signaling by growth factors and cytokines [23]. For example, NADPH oxidase activity plays defensive roles in phagocytic cells [18]. The small size and ability of ROS (such as H_2_O_2_) to traverse membranes make them suitable for cell signaling; however, this role is not yet fully understood in *Plasmodium* [24].

### 3.2. Lipid Peroxidation

**Figure 3:**
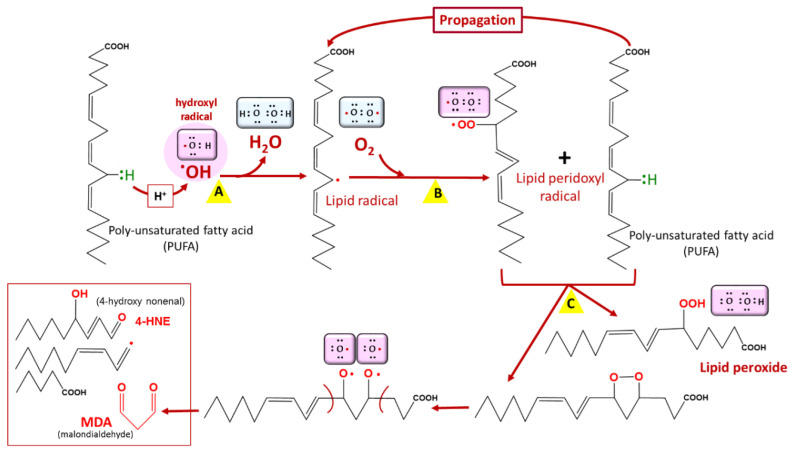
Lipid peroxidation. Lipid peroxidation occurs in three phases: initiation (A), propagation (B), and termination (C). Malondialdehyde (MDA) is a biomarker of lipid peroxidation in living cells. Among lipids, polyunsaturated fatty acids (PUFAs) are the most vulnerable to lipid peroxidation. COOH = carboxyl group, OOH = hydroperoxyl.

Lipids are prone to attacks from reactive species, resulting in their oxidation and the formation of lipid peroxides [25] (Figure 3). This leads to cellular dysfunction, especially as lipids are major components of cell membranes. In *Plasmodium*, artemisinin and its derivatives accumulate in neutral lipid bodies, especially in the digestive vacuole, where they can trigger oxidative damage after their heme–iron activation [26], via a lipid peroxidation process that, once initiated, is propagated by autocatalysis to free fatty acids [26]. This oxidative damage leads to a loss of Plasmodium membrane integrity and, consequently, parasite death. Moreover, tetraoxanes oxidatively damage phospholipids more than artemisinin [27], which may account for the differential antimalarial effect of these endoperoxides.

### 3.3. Protein Damage

**Figure 4:**
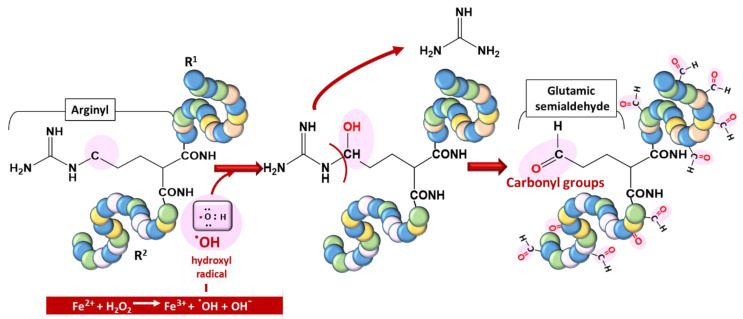
Typical carbonylation of proteins based on a metal-catalyzed oxidation (MCO) attack involving a transition metal (Fe^2+^) for hydroxyl radical generation.

Proteins are vulnerable to oxidation by ROS, a phenomenon generally referred to as protein carbonylation. Protein carbonyls are reactive aldehydes and ketone adducts formed through the α-amidation pathway, the formation of protein–protein cross-linked derivatives, the oxidative cleavage of glutamyl residues, and cell membrane damage by lipid oxidation products [28]. Protein carbonyls have been used as biomarkers of oxidative stress due to their relative stability and early formation [29]. ROS generate cytotoxic protein carbonyls in two main and irreversible ways:

(i) by a metal-catalyzed oxidative (MCO) attack targeting the amino acid moiety of arginine, lysine, threonine, and proline (Figure 4);
(ii) by secondary reactions on lysine, cysteine, and histidine with reactive carbonyl derivatives, resulting, among other things, from lipid peroxidation.

These post-translational modifications due to the oxidization of amino acid side chains, which results in aldehyde, ketone, and lactam formation, can also lead to damage to proteins [30,31].

The endoperoxides alkylate numerous vital proteins, including enzymes in the parasite, particularly the cysteine residue of cysteine proteases, which play a vast role in *P. falciparum*, ranging from hemoglobin (Hb) uptake and digestion to aiding red blood cell rupture [8,32,33]. Therefore, their alteration, leading to the inhibition of their enzymatic properties, can cause severe setbacks for parasite survival, considering the huge dependence of the parasite on Hb digestion products. Endoperoxides also disrupt the activities of sarcoplasmic–endoplasmic reticulum Ca^2+^-ATPase- (SERCA-) type protein, encoded by the *pfatpase6* gene, presumably from the ROS that they generate [34]. More recently, approximately 124 proteins were identified as covalent binding targets of artemisinins [35].

### 3.4. Nucleic Acid Damage

**Figure 5:**
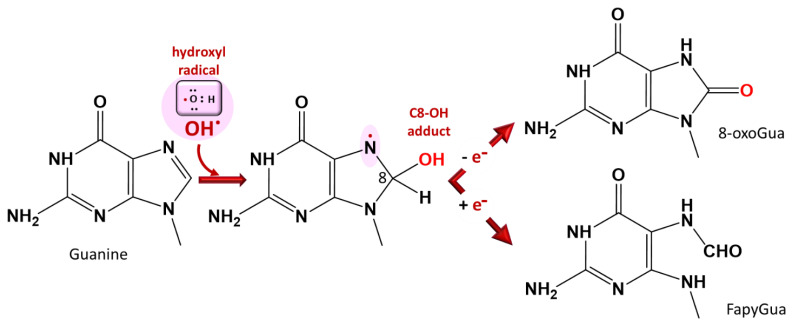
DNA base damage by ROS at the purine base guanine. The C8-OH adduct 7-hydro8-oxo-2’-deoxyguanosine is used as a marker of oxidative stress. A similar reaction occurs with adenine (8-oxoAde and Fapy-Ade). FapyGua: formamidopyrimidine, 8-oxoGua: 8-oxoguanine [36,39].

The molecular integrity of DNA and other genetic material is necessary for the continued existence and survival of all living organisms, including *Plasmodium*. ROS cause structural damage to DNA by attacking mainly one of its bases, guanine, due to its lower oxidation potential [36,37] (Figure 5). Reports have revealed that artesunate can cause double-stranded breaks of plasmodial DNA in less than one hour and that it is linked to an increase in ROS generation [38].

Disruption of the conformation of biomolecules by artemisinins (endoperoxides) or other ROS-generating antimalarials kills parasites. Although they have a similar activation process generating carbon- and oxygen-centered radicals, the efficacy of endoperoxides varies, which can be explained, in part, by their different pharmacological properties, notably the stage of the parasite erythrocyte cycle, the nature of their target, and their location in the parasite, i.e., trioxolanes vs. artemisinin and its derivatives [26,40].

## 4. Sources and Management of ROS in *Plasmodium*-Infected Erythrocytes under Steady State

ROS arise from both the metabolism of the parasite and the host defense system [41]. In erythrocytic parasites, there are two important sources of ROS: the mitochondrial electron transport chain and the degradation of hemoglobin [24,42]. The involvement of the antioxidant machinery and the elimination of heme produced during the digestion of hemoglobin are the two strategies by which the parasite maintains its redox homeostasis (Table 1).

### 4.1. ROS Production from Mitochondrial Electron Transport Chain

The mitochondrion constitutes a very important source of free radicals in aerobic organisms. Approximately 1–3% of electrons escape from the electron transport chain directly to react with molecular oxygen, leading to the formation of superoxide radicals (O_2_^•−^) [15]. This is all the more important as the mitochondrion of the parasite responsible for malaria has cytochromes with heme as a cofactor in the electron transport chain [51]. Although there is a paucity of data on the involvement of the parasitic mitochondrion in ROS generation, the complexity and diversity of its antioxidant system is a very good pointer, especially the report of the expression of cytosolic PfSOD-1 and mitochondrial PfSOD-2 throughout the blood stages of the parasite [52]. Superoxide dismutases (SODs) quickly dismutate the formed superoxide radical (O_2_^•−^) to hydrogen peroxide (H_2_O_2_), which can be reduced to water by the peroxiredoxin 2-Cys Prx (TPx-2) [53,54].

### 4.2. ROS Production from Hemoglobin Digestion

**Figure 6:**
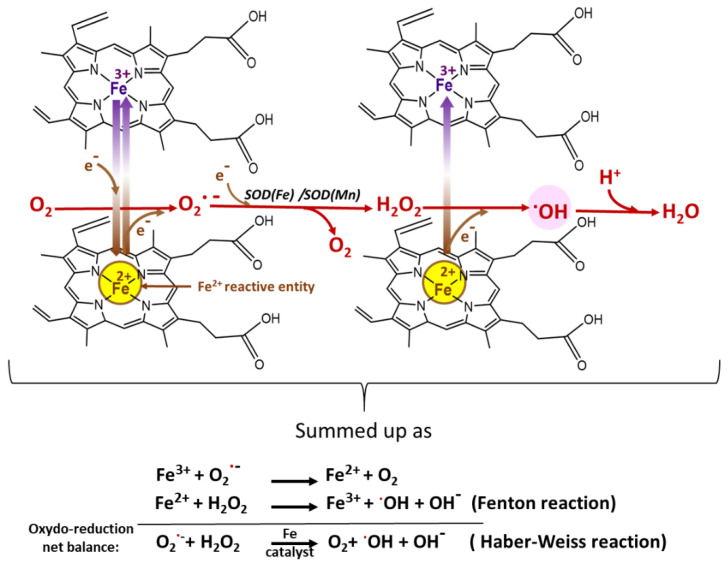
Heme Fe^2+^ reaction with O_2_^•−^ leading to ROS production, especially the most deleterious one, the hydroxyl radical ^•^OH [3,63].

During the blood stage, parasites take up and break down approximately 75% of the hemoglobin (Hb) of red blood cells to obtain essential amino acids for their development and replication, releasing free heme as residual toxic waste [44,55]. This free heme contains reactive ferrous iron (Fe^2+^) that can readily reoxidize by transferring electrons to oxygen to form superoxide radicals (O_2_^•−^) [16] and then hydrogen peroxide, finally leading to the production of the highly deleterious hydroxyl radical (^•^OH) through the Fenton reaction involving a new molecule of heme (Fe^2+^) (Figure 6) [3].

**Figure 7:**
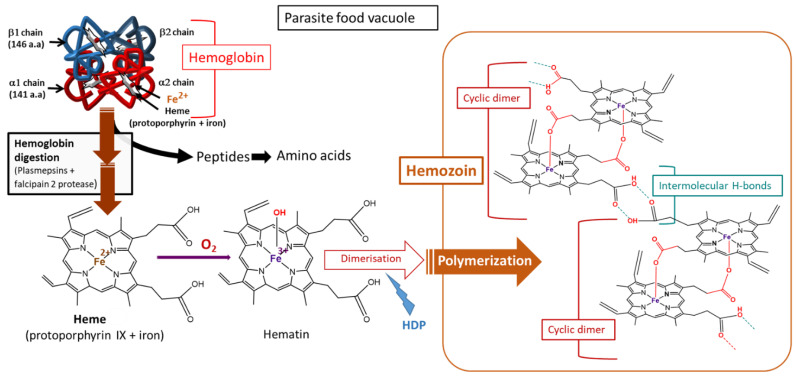
Hemoglobin digestion and hemozoin production in *Plasmodium*-infected red blood cells. HDP, heme detoxification protein [59,64].

Because of the important role that heme plays in ROS generation, immediately after its production, a detoxification process takes place where approximately 95% of the produced heme (Fe^2+^) is polymerized into hemozoin, a nontoxic crystal [46]. This reaction involves the heme detoxification protein (HDP), whose action is aided by histidine-rich protein-2 (HRP2) (Figure 7) [48,56]. HRP2 is said to have a very high affinity for heme [57,58], and elevated HRP2 reduces the vulnerability of the parasite. Kapishnikov et al. showed that mature parasites have approximately 70% of the total iron from red blood cells in the hemozoin crystals and therefore suggested a coupling of the rate of Hb digestion to that of heme polymerization [59].

Although *Plasmodium* lacks the heme oxygenase enzyme, which is deployed by most organisms to degrade heme [60], it has a system in place that mimics the heme oxygenase [44]. During the digestion of hemoglobin, H_2_O_2_ is also generated in the food vacuole as a result of the immediate conversion of oxyhemoglobin to methemoglobin due to heme reduction at the prevailing pH of 5.2 [61,62]. The heme is peroxidatively degraded by the reaction with H_2_O_2_, leading to the formation of a ferryl intermediate (Fe(IV)=O) [44].

*Plasmodium* uses a family of hemoglobinases to degrade hemoglobin into amino acids. *Plasmodium* also requires heme from the host to synthesize proteins such as the heme-dependent cytochromes in the mitochondrial electron transport chain [51], although genetic studies have revealed that the parasites encode and express all the enzymes for heme production [60]. Moreover, the digestion of hemoglobin ensures that the infected red blood cells are osmotically stable throughout the intraerythrocytic stages of the parasite [65]. The digestion of hemoglobin is therefore essential for *Plasmodium* during its intraerythrocytic cycle. In addition to the formation of hemozoin, there are other antioxidant defense mechanisms (discussed below) that can address the toxic effect of resting unpolymerized heme. The significant role played by hemoglobin in oxygen transport makes red blood cells and their vicinity highly vulnerable to ROS formation [66].

Box 1 **The role of nitric oxide in potentiation of reactive species [15,67,68].**

**Potentiation of reactive species: Nitric oxide in view**

Nitric oxide (^•^NO), which is also considered a ROS, is produced by macrophages against *Plasmodium*. The free radical nitric oxide reacts with the primary radical, O_2_^•−^, to give peroxynitrite, a more lethal reactive species, which can also damage biomolecules such as proteins and DNA. It remains to be seen if ^•^NO, produced by human phagocytes, diffuses into the parasitic cytosol. However, ^•^NO production has also been reported in the parasite by an isoform of nitric oxide synthase (NOS), which is calcium-independent and whose inhibition affects the growth of *P. falciparum*. The ^•^NO production is higher in the trophozoite than in the ring stage. Although ^•^NO may not be very deleterious, its reaction with H_2_O_2_ to form peroxynitrite will potentiate their radicality

### 4.3. Management of ROS by Plasmodium under Steady State: The Antioxidant Machinery

To match the multiplicity of the sources of ROS, including nitric oxide (Box 1), from both the host and the parasites themselves, malaria parasites have several antioxidant machineries in addition to heme elimination processes, which may be additive or synergistic in ensuring their survival in red blood cells. *Plasmodium* species express antioxidant proteins/enzymes such as superoxide dismutases (SODs), glutathione/glutathione-dependent proteins, thioredoxin/thioredoxin-dependent proteins and thioredoxin reductase, and peroxiredoxins [53,69,70,71].

**Figure 8:**
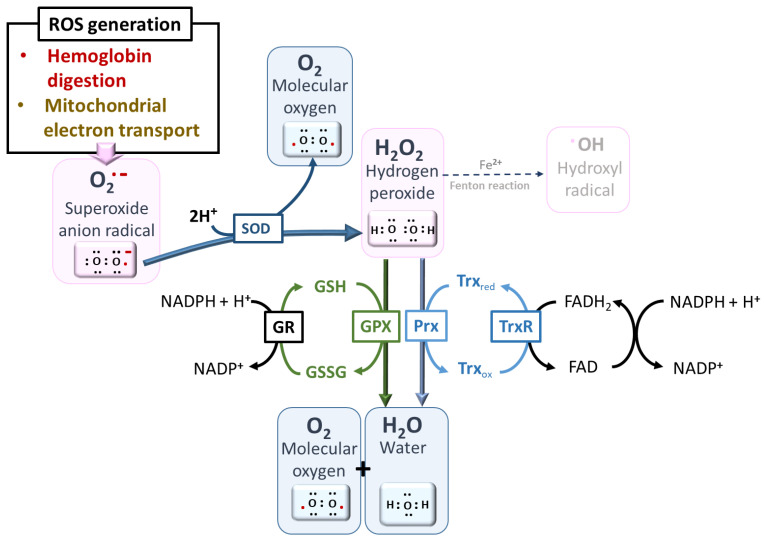
Major pathways of ROS detoxification. A network of redox cycling cofactors, such as flavin adenine dinucleotide (FADH2) and nicotinamide adenine dinucleotide phosphate (NADPH), aids in the reduction of ROS, avoiding the ultimate and deleterious production of hydroxyl radical ^•^OH from H_2_O_2_ by the Fenton reaction in the presence of reduced iron [52]. Abbreviations—SOD: superoxide dismutase, GR: glutathione reductase, GPX: glutathione peroxidases, Prx: peroxiredoxins, TrxR: thioredoxin reductase, GSH: reduced glutathione, GSSG: oxidized glutathione dipeptide, Trx_red_: reduced thioredoxin, Trx_ox_: oxidized thioredoxin.

- The SODs act fundamentally to enable the spontaneous dismutation of O_2_^•−^ to H_2_O_2_, which can then be reduced by other enzymes to water, preventing its reaction with iron (Fe^2+^) to form highly toxic ^•^OH. *Plasmodium* expresses two forms of SOD: cytosolic PfSOD-1 (Fe-SOD) and mitochondrial PfSOD-2 (Mn-SOD). The uptake of SOD from the host may also be complementary [52,72].
- Glutathione, a tripeptide composed of cysteine, glycine, and glutamate, plays a vital role in the defense system of malaria parasites. Although the level of heme that escapes polymerization is only 5%, it can still mount oxidative stress, which can be eliminated by GSH-dependent mechanisms [45,46,73]. GSH is generated by the reduction of GSSG by glutathione reductase, which can be amplified by other proteins with thiol groups, such as thioredoxin (Trx) [74] (Figure 8). This could be further boosted by the de novo synthesis of GSH, a predominant pathway under oxidative stress [75,76]. Suggestions of possible uptake from the host were rebuffed by the findings of Patzewitz et al., who demonstrated that GSH uptake from the host is not statistically significant [77]. In addition to the nonenzymatic degradation of heme by GSH to generate nonheme iron, *Plasmodium* also possesses a thiol enzyme, glutathione-S-transferase (GST), which binds and sequesters heme (Fe^2+^) [78]. To affect its detoxification roles, GSH also serves as a cofactor for GSH-dependent enzymes such as GST, which helps in the reduction of H_2_O_2_ and glutathione-peroxidase-like proteins. Overall, GSH acts as a redox buffer to provide redox homeostasis [79]. This is essential in preventing the escalation of ROS generation, which is often aided by the redox cycling of transition metals such as iron.
- Thioredoxins (Trxs) are disulfide oxidoreductases, expressed in all organisms, that interact with a myriad of proteins through their cysteine moiety to donate electrons [80,81]. Trxs keep biomolecules in their reduced and active conformations, consequently promoting parasite survival under oxidative stress. The regeneration of reduced thioredoxin is achieved through the action of thioredoxin reductase (a FAD-dependent enzyme) using NADPH [82]. Three isoforms of Trx have been characterized: a cytosolic isoform, Trx1, and two isoforms in the apicoplast, Trx2 and Trx3 [50].
- *Plasmodium* lacks catalase and glutathione peroxidase and therefore will likely depend greatly on peroxiredoxins (Prx) to reduce H_2_O_2_ generated by SOD [52,53]. *P. falciparum* expresses five different isoforms of Prx that are strategically localized in the cytosol, mitochondria, and perhaps the apicoplast. These are 1-Cys Prx, two typical 2-Cys Prxs (Prx1 and Prx2), a 1-Cys antioxidant protein (AOP), and a GSH peroxidase-like thioredoxin peroxidase (TPx_Gl_) [50]. It is known that 1-CysPrx and Prx1 are localized in the cytosol, while Prx2 is localized in the mitochondria. The localizations of AOP and TPx_Gl_ are not yet fully understood. Prx1, one of the most abundant peroxidases, is constitutively expressed across all blood parasitic stages and has a very high affinity for H_2_O_2_, hence being judged to serve a housekeeping role [53,83,84]. Prxs are kept in reduced form through reduction by thioredoxin-dependent systems, comprising three Trxs and Trx-like proteins (TLPs), Tlp1 and Tlp2, which have been identified genomically in the parasite [50]. The parasite, in addition to the expressed Trxs, has approximately 50% of its thioredoxin peroxidase activity depending on the imported human Prx-2 (hPrx-2) [85]. Moreover, it has been demonstrated that H_2_O_2_ can diffuse into the host and be handled by the host’s catalase enzyme [86]. The import of human antioxidant machinery may be applicable to other antioxidants, although it has not been fully verified.
- NADPH is involved in maintaining vital antioxidant enzymes, such as glutathione and thioredoxin reductases, in their active conformations. However, the accumulation of heme shifts the equilibrium toward the oxidized form NADP+, increasing the pressure on the reduced form NADPH necessary to reduce the oxidized glutathione (GSSG) to glutathione (GSH). This can lead to the suppression of the main antioxidant mechanisms, such as glutathione reductase, thioredoxin reductase (TrxR), glyceraldehyde-3-phosphate dehydrogenase, and other proteins, leading to the elevation of oxidative stress [87].
- Vitamin B6, in addition to playing several biochemical roles, has also been seen to act as a potent antioxidant [88,89]. The enzymes governing vitamin B6 synthesis are elevated in parasites exposed to oxidative stress, e.g., generated by methylene blue and hence seen as a potential pharmacological target [49]. Although its antioxidant mechanism is still subject to debate, it is believed to scavenge ROS and prevent lipid peroxidation [90,91,92].

## 5. ROS Production in *Plasmodium*-Infected Erythrocytes under Antimalarial Treatment

Oxidative stress is not only an important clinical and pathobiochemical factor but also an effective therapeutic principle in malaria. Indeed, the pharmacological activity of some of the most important antimalarials, such as quinolines, atovaquones, and artemisinin derivatives, is mediated by the high production of ROS, exceeding the oxidative stress management capacities of the parasite [16,93].

### 5.1. Mode of Action of Chloroquine and Other Quinolines

Quinolines have been reported to inhibit the polymerization of heme to hemozoin in food vacuoles, leading to the accumulation of heme [94]. Moreover, chloroquine and other quinolines differently inhibit the peroxidative degradation of heme by H_2_O_2_, hence increasing heme accumulation and consequently enhancing heme-catalyzed reactions, leading to the production of ROS and parasite death [44,95]. The formation of the heme–Fe^III^ adduct with chloroquine in the food vacuole leads to its diffusion into the cytosol, where the complex is dissociated, releasing heme in the parasite cytosol and hence promoting the redox cycling of Fe^III^ to Fe^II^, which enhances ROS generation [96]. This has also been reported for quinine [96] and may apply to other quinolines. Other purported modes of action of quinolines include the inhibition of the peroxidase-like activities of heme, leading to the accumulation of H_2_O_2_, which interacts with heme to form radicals [97]. Reviewed reports about the mode of action of quinolines have revealed their varying levels of activity that trigger ROS generation [44,94,96].

### 5.2. Mode of Action of Atovaquone and Hydroxynaphtoquinones

The mode of action of atovaquone and other hydroxynaphtoquinones is based on the inhibition of mitochondrial cytochrome *bc1* complex via the competitive inhibition of ubiquinol binding [98,99]. Consequently, atovaquone induces the collapse of the mitochondrial membrane potential, blocking the energy supply of the parasites, leading to parasite death [100,101]. Concomitantly, the ubiquinol accumulation due to the inhibition of its binding site generates also a significant amount of superoxide radicals (ROS), as was shown in cancer cells and *Plasmodium* [16,102]. 

### 5.3. Mode of Action of Artemisinin: The Role of Endoperoxide 

Artemisinins are at the forefront of the war against malaria and are used in combination with other antimalarial agents in artemisinin-based combination therapies (ACTs) [103,104]. This class of compounds, which includes arteether, artemether, artesunate, and dihydroartemisinin [105], is characterized by the presence of an endoperoxide group that is responsible for the antimalarial activity.

#### 5.3.1. Activation of the Endoperoxide to Generate ROS

**Figure 9:**
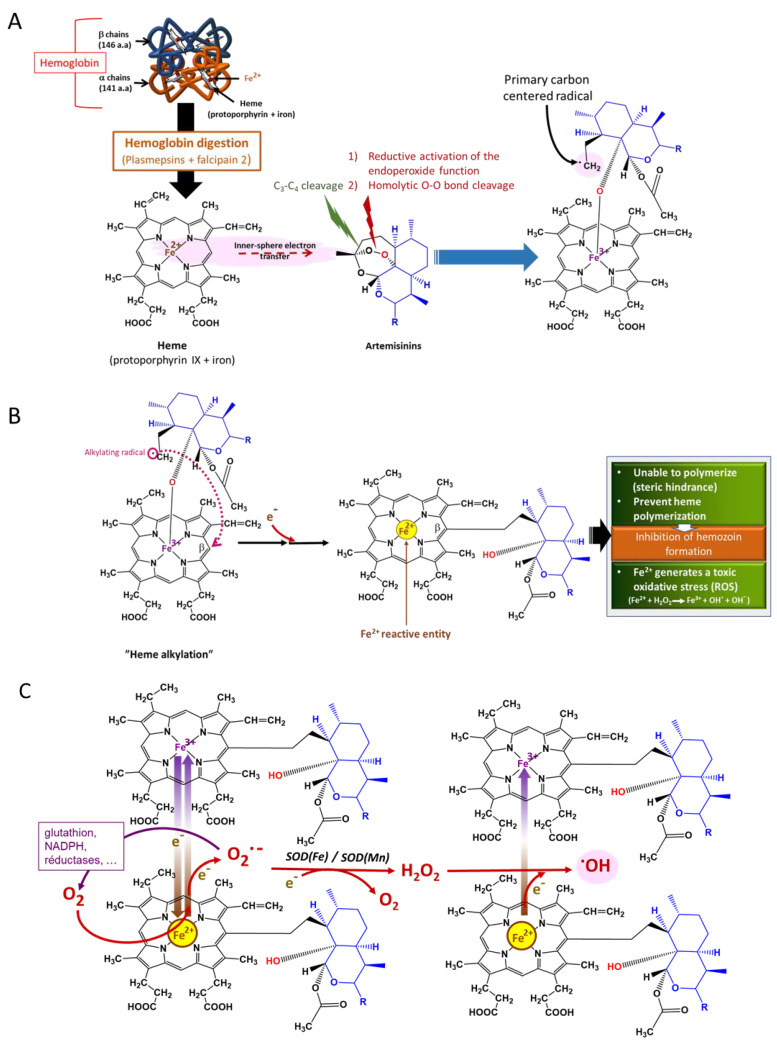
Mode of action of artemisinins [109]. (**A**) Primary carbon-centered radical formation; (**B**) heme alkylation and adduct formation of heme–artemisinin; (**C**) ROS generation, especially the most deleterious hydroxy radical, •OH.

The endoperoxides are converted into carbon-centered radicals, which mediate most of their effects. Activation is performed by the transfer of electrons from transition metals, mainly iron (Box 2), leading to the homolytic cleavage of the endoperoxide bridge [106,107,108,109]. The carbon-centered radical formed can alkylate heme, preventing its polymerization into hemozoin and thus inducing the formation of toxic ROS. They also alkylate several essential biomolecules, rendering them dysfunctional and consequently leading to plasmodial death. The iron could be heme (nonchelatable iron) (Figure 9) or freely circulating iron (chelatable iron).

Available evidence shows that artemisinin reacts more efficiently with heme than other forms of iron [110], thus pointing to the huge role played by heme in the pharmacological activity of endoperoxides. The dependence on iron is affected by the stage of the parasite. However, artemisinin activation at the early ring stages depends more on chelatable iron than on the trophozoite stage (35% vs. 15%), for which artemisinin activation depends more on heme generation from hemoglobin digestion [111]. This could be explained by a hemoglobinase system that is not fully developed at the ring stage but has a low level of falcipain 2 and 3 activity sufficient to sustain hemoglobin degradation that permits normal growth [111]. The molecular structure of an endoperoxide also determines its level of dependence on heme for activation [111]. Although the most studied source of heme for artemisinin activation is hemoglobin, the debate continues on whether other heme-containing proteins may not do the same [107,112]. Some of these proteins are cytochrome p450, catalases, peroxidases, and other heme-containing proteins [113,114,115,116]. The use of chelators of free iron and inhibitors of hemoglobin digestion revealed that artemisinin activity at the early ring and trophozoite stages depends more than 80% on heme activation, while, at the mid-ring stage, it is 40% dependent. This means that artemisinin and, by extension, other endoperoxides may have other mechanisms of action that may not be iron-dependent [111].

#### 5.3.2. Depolarization of the Mitochondrial Membrane Potential

Mitochondria can play an essential role in the indirect killing of the parasite from ROS generation [107,112]. ROS production occurs as a result of membrane depolarization, dissipating its membrane potential, which plays vital roles in maintaining parasite cellular integrity, hence causing death [117,118]. This depolarization can be caused by the direct inhibition of PfATPase6 by artemisinins and other endoperoxides [119]. Artemisinins can also generate ROS at the mitochondrial level by altering the transfer of electrons from complex III to molecular oxygen, forming superoxide radicals [16,38,69,112].

Box 2 **Probable roles of other transition metals as cofactors in endoperoxide activities [15,96].**

**Transition metals potentiate the artemisinin activity**

Apart from iron, other metals, especially transition metals such as copper, which are essential components of some proteins, also play a crucial role in ROS generation in the malaria parasite by catalyzing redox cycling in their unbound forms. The ROS generation by antimalarials is potentiated through the redox recycling of Fe^3+^ to Fe^2+^ with the aid of reduced flavin cofactors and probably NADPH-Fe (flavin reductase). Among the transition metals are copper and zinc.

### 5.4. Artemisinin-Based Combination Therapies

As discussed earlier, quinolines and artemisinins act essentially via the production of ROS. The WHO has recommended six artemisinin-based combination therapies (ACTs): artemether–lumefantrine (AL), dihydroartemisinin–piperaquine (DHA-PPQ), artesunate–amodiaquine (AS-AQ), artesunate–mefloquine (AS-MQ), artesunate–sulfadoxine-primethamine (AS-SP), and artesunate–pyronaridine (AS-PY) [120]. ACTs drastically clear the parasite and resolve malaria symptoms (such as fever) [121], hence the need for their continued use. Synthetic hybrids of endoperoxides and quinolines have been developed to enhance their pharmacological activities and cost-effectiveness in malaria treatment [105,122]. Resistance to partner drugs used in ACTs in Southeast Asia raises concern over the long-term usefulness of these antiplasmodial combinations [120,123,124].

### 5.5. ROS Evasive Mechanisms under Treatment

To handle the exacerbated ROS generation by the presence of antimalarials, the parasites upregulate their survival mechanisms, in addition to the already discussed mechanisms. The parasite’s evasive mechanisms could simply be classified as preventive, reductive, and reparative.

#### 5.5.1. Preventive Mechanisms

Reduction of self-generated ROS: The parasite is said to reduce its own production of ROS and develop new evolutionary mechanisms to curb the effect of ROS [125]. This evolutionary mechanism undergoes apicoplast metabolism with lipoic acid production, which has antioxidant properties. Lipoic acids are disulfide-containing derivatives of octanoic acid that can exist in oxidized and reduced forms. This ability enables them to play antioxidant roles. Lipoic acids serve as cofactors to several enzymes involved in energy and amino acid metabolism [126]. In the case of artemisinin resistance, reduced hemoglobin import leads to a reduction in the activation of artemisinin and reduced ROS generation [127].

Reduced or mutated expression of hemoglobinases and reduced Hb endocytosis: Endoperoxide activation largely depends on heme production from Hb [110,111]. Downregulation of hemoglobinases (falcipain 2 and 3) in the early ring stage could participate in artemisinin resistance by enhancing the effect of the K13 mutation [111]. Moreover, it has been established that a reduction in the import of hemoglobin, especially at the ring stage, in K13 mutants is responsible for artemisinin resistance [127,128]. Quiescence state was also described as a parasite response to face the oxidative stress generated by artemisinin [129,130,131]. The parasites recuperate rapidly from dormancy artemisinin-induced once the treatment is removed [130,131,132].

#### 5.5.2. Reductive Mechanisms

***Import of biomolecules***: The host boasts more developed antioxidant machinery against ROS than *Plasmodium*. Regardless of this, *Plasmodium* can develop evolutionary measures to eliminate the marauding ROS in its cytosol. Among these measures is the importation of some human antioxidant machinery, such as human peroxiredoxin-2, which uses PfTrx as a reducing agent. Importation is reported to be elevated under treatment with ROS-generating antimalarials [85]. Other forms of human proteins (SOD, catalase, aminolevulinic acid dehydratase, and ferrochelatase) that may augment parasitic defense mechanisms have also been reportedly imported by *Plasmodium* [72,133,134,135].

***Increased expression of antioxidants:*** Because of the elevated ROS generation due to antimalarial treatment, parasites increase their expression of vital antioxidant enzymes. Such enzymes include iron-superoxide synthetase (Fe-SOD), glutathione-S-transferase (GST), glutathione synthetase (GS), γ-glutamylcysteine synthetase (γ-GCS), thioredoxin reductase (TrxR), and peroxiredoxins (nPrx) [69,70,136]. This overexpression, which is higher in the artemisinins-resistant strains, especially SOD and GST, is associated with parasite resistance characterized by a lower level of ROS and less oxidized proteins [69].

#### 5.5.3. Reparative Mechanisms

ROS generated by antimalarials damage many biomolecules, such as proteins, lipids, and nucleic acids [15]. Increased repair is therefore necessary for continued survival under elevated ROS production. The unfolded protein response (UPR), often referred to as a stress gene, is essential for parasite survival and is upregulated in K13 mutants. This confers upon the parasite an increased ability to repair or degrade proteins damaged by alkylation and oxidation generated by artemisinin [137]. Moreover, K13 mutations have been associated with the elevation of *P. falciparum* phosphatidylinositol-3-kinase (PfPI3K) due to its reduced association with PfKelch13 and polyubiquitination [138]. This consequently leads to the elevation of phosphatidylinositol-3-phosphate (PI3P), which is said to be essential in the trafficking of proteins and lipids toward the apicoplast, where they are needed [138,139]. K13 gene mutations have demonstrated a global spread and attracted increased attention, especially in endemic regions, as molecular markers for ART resistance [129], although not all have been linked to resistance to artemisinin [140,141]. Single-nucleotide polymorphisms (SNPs) in *P. falciparum mlh1*, *pms1*, and *exo1* lead to increased expression of thioredoxin (PfTrx) and signal peptide peptidase, which increases adaptation to oxidative stress and protein damage, leading to the upregulation of the DNA repair mechanisms of the parasite with a consequent decrease in the antimalarial effect of artemisinin [136,142]. These mechanisms keep the vital biomolecules of parasites functional and, as a result, improve their chances of survival under treatment, i.e., resistance development.

## 6. Conclusions

It is now apparent that conventional antimalarials deploy ROS, ultimately responsible for their parasiticidal activity. However, the evolutionary dynamism of *Plasmodium* seeks to systematically counteract the antimalarial activity mediated by ROS. Given this observation, it is unsurprising that the time required for *P. falciparum* to acquire resistance to antimalarial drugs, whose mode of action is based on the generation of ROS, is becoming increasingly shorter. In this way, the development of artemisinin resistance to new endoperoxide-based hybrid molecules indicates a shared pathway of resistance to the K13 propeller gene mutation [143]. To ensure the continued relevance of ROS-producing antimalarials, the ROS-managing machinery of the parasite could be thwarted to preserve and enhance the activities of the antimalarials. ACTs with newer molecules that disrupt the resistance mechanism of the parasite should be sought. Such molecules, companion drugs of artemisinin, could target key antioxidant enzymes linked to glutathione-dependent and thioredoxin-dependent systems, phosphatidyl inositol 4-kinase enzyme (PI4K), and mRNA translation pathways in *Plasmodium* [69,144,145,146,147]. ROS generation thus remains a key element in the strategy in the fight against *Plasmodium* and sustainable malaria control.

## Figures and Tables

**Table 1:** Plasmodial heme elimination and antioxidant machinery.

Agent	Site of Production	Role in Oxidative Homeostasis
HRP	FV	Binding with heme for polymerization
HDP	FV	Heme polymerization to hemozoin
H_2_O_2_	Cytosol and FV	Degrades heme
SOD	Cytosol, Mitochondria	Dismutation of O_2_^•−^
Prx	Cytosol, Mitochondria, Apicoplast	Reduction of H_2_O_2_ to H_2_O
Trx	Cytosol, Mitochondria	Reduction of Prx
GSH	Cytosol	Degradation of heme, reduction of proteins and ROS
Vit B6	Cytosol	Role unclear

H_2_O_2_: hydrogen peroxide, HRP: histidine-rich protein, HDP: heme detoxification protein, SOD: superoxide dismutase, Trx: thioredoxin, Prx: peroxiredoxin, GSH: reduced glutathione, Vit B6: vitamin B6, FV: food vacuole [43,44,45,46,47,48,49,50].

## References

[1] WHO World Malaria Report 2020: 20 Years of Global Progress and Challenges. https://www.who.int/teams/global-malaria-programme/reports/world-malaria-report-2020.

[2] Bilgin R., Yalcin M.S., Yucebilgic G., Koltas I.S., Yazar S. (2012). Oxidative stress in vivax malaria. Korean J. Parasitol..

[3] Becker K., Tilley L., Vennerstrom J.L., Roberts D., Rogerson S., Ginsburg H. (2004). Oxidative stress in malaria parasite-infected erythrocytes: Host-parasite interactions. Int. J. Parasitol..

[4] Erel O., Vural H., Aksoy N., Aslan G., Ulukanligil M. (2001). Oxidative stress of platelets and thrombocytopenia in patients with vivax malaria. Clin. Biochem..

[5] WHO (2019). World Malaria Report 2019.

[6] Sahu M., Tediosi F., Noor A.M., Aponte J.J., Fink G. (2020). Health systems and global progress towards malaria elimination, 2000–2016. Malar. J..

[7] Dako-Gyeke M., Kofie H.M. (2015). Factors influencing prevention and control of malaria among pregnant women resident in Urban slums, Southern Ghana. Afr. J. Reprod. Health.

[8] Thomas J.A., Tan M.S.Y., Bisson C., Borg A., Umrekar T.R., Hackett F., Hale V.L., Vizcay-Barrena G., Fleck R.A., Snijders A.P. (2018). A protease cascade regulates release of the human malaria parasite Plasmodium falciparum from host red blood cells. Nat. Microbiol..

[9] Yewhalaw D., Wassie F., Steurbaut W., Spanoghe P., Van Bortel W., Denis L., Tessema D.A., Getachew Y., Coosemans M., Duchateau L. (2011). Multiple Insecticide Resistance: An Impediment to Insecticide-Based Malaria Vector Control Program. PLoS ONE.

[10] Shibeshi M.A., Kifle Z.D., Atnafie S.A. (2020). Antimalarial Drug Resistance and Novel Targets for Antimalarial Drug Discovery. Infect. Drug Resist..

[11] Payne D. (1987). Spread of chloroquine resistance in Plasmodium falciparum. Parasitol. Today.

[12] Noedl H., Se Y., Schaecher K., Smith B.L., Socheat D., Fukuda M.M. (2008). Evidence of artemisinin-resistant malaria in Western Cambodia. N. Engl. J. Med..

[13] Dondorp A.M., Nosten F., Yi P., Das D., Phyo A.P., Tarning J., Lwin K.M., Ariey F., Hanpithakpong W., Lee S.J. (2009). Artemisinin Resistance in Plasmodium falciparum Malaria. N. Engl. J. Med..

[14] Li Y.R., Trush M. (2016). Defining ROS in Biology and Medicine. React. Oxyg. Species.

[15] Santo A., Zhu H., Li Y.R. (2016). Free Radicals: From Health to Disease. React. Oxyg. Species.

[16] Egwu C.O., Tsamesidis I., Pério P., Augereau J.-M., Benoit-Vical F., Reybier K. (2021). Superoxide: A major role in the mechanism of action of essential antimalarial drugs. Free Radic. Biol. Med..

[17] Mohring F., Pretzel J., Jortzik E., Becker K. (2014). The redox systems of Plasmodium falciparum and Plasmodium vivax: Comparison, in silico analyses and inhibitor studies. Curr. Med. Chem..

[18] Hancock J.T., Desikan R., Neill S.J. (2001). Role of reactive oxygen species in cell signalling pathways. Biochem. Soc. Trans..

[19] Mittal M., Siddiqui M.R., Tran K., Reddy S.P., Malik A.B. (2014). Reactive Oxygen Species in Inflammation and Tissue Injury. Antioxid. Redox Signal..

[20] Stefanatos R., Sanz A. (2018). The role of mitochondrial ROS in the aging brain. FEBS Lett..

[21] Sugamura K., Keaney J.F. (2011). Reactive oxygen species in cardiovascular disease. Free Radic. Biol. Med..

[22] Deavall D.G., Martin E.A., Horner J.M., Roberts R. (2012). Drug-induced oxidative stress and toxicity. J. Toxicol..

[23] Thannickal V.J., Fanburg B.L. (2000). Reactive oxygen species in cell signaling. Am. J. Physiol. Lung Cell. Mol. Physiol..

[24] Rahbari M., Rahlfs S., Jortzik E., Bogeski I., Becker K. (2017). H2O2 dynamics in the malaria parasite Plasmodium falciparum. PLoS ONE.

[25] Sampson C., Keens R.H., Kattnig D.R. (2019). On the magnetosensitivity of lipid peroxidation: Two- versus three-radical dynamics. Phys. Chem. Chem. Phys..

[26] Hartwig C.L., Rosenthal A.S., D’Angelo J., Griffin C.E., Posner G.H., Cooper R.A. (2009). Accumulation of artemisinin trioxane derivatives within neutral lipids of Plasmodium falciparum malaria parasites is endoperoxide-dependent. Biochem. Pharmacol..

[27] Kumura N., Furukawa H., Onyango A.N., Izumi M., Nakajima S., Ito H., Hatano T., Kim H.S., Wataya Y., Baba N. (2009). Different behavior of artemisinin and tetraoxane in the oxidative degradation of phospholipid. Chem. Phys. Lipids.

[28] Fernando N., Wickremesinghe S., Niloofa R., Rodrigo C., Karunanayake L., De Silva H.J., Wickremesinghe A.R., Premawansa S., Rajapakse S., Handunnetti S.M. (2016). Protein carbonyl as a biomarker of oxidative stress in severe leptospirosis, and its usefulness in differentiating leptospirosis from dengue infections. PLoS ONE.

[29] Dalle-Donne I., Rossi R., Giustarini D., Milzani A., Colombo R. (2003). Protein carbonyl groups as biomarkers of oxidative stress. Clin. Chim. Acta.

[30] Nyström T. (2005). Role of oxidative carbonylation in protein quality control and senescence. EMBO J..

[31] Fedorova M., Bollineni R.C., Hoffmann R. (2014). Protein carbonylation as a major hallmark of oxidative damage. Mass Spectrom Rev.

[32] Rosenthal P.J. (2004). Cysteine proteases of malaria parasites. Int. J. Parasitol..

[33] Wu W.M., Chen Y.L., Zhai Z., Xiao S.H., Wu Y.L. (2003). Study on the mechanism of action of artemether against schistosomes: The identification of cysteine adducts of both carbon-centred free radicals derived from artemether. Bioorg. Med. Chem. Lett..

[34] Eckstein-Ludwig U., Webb R.J., Van Goethem I.D.A., East J.M., Lee A.G., Kimura M., O’Neill P.M., Bray P.G., Ward S.A., Krishna S. (2003). Artemisinins target the SERCA of Plasmodium falciparum. Nature.

[35] Wang J., Xu C., Lun Z.R., Meshnick S.R. (2017). Unpacking ‘Artemisinin Resistance’. Trends Pharmacol. Sci..

[36] Jena N.R. (2012). DNA damage by reactive species: Mechanisms, mutation and repair. J. Biosci..

[37] Cadet J., Wagner J.R. (2013). DNA base damage by reactive oxygen species, oxidizing agents, and UV radiation. Cold Spring Harb. Perspect. Biol..

[38] Gopalakrishnan A.M., Kumar N. (2015). Antimalarial action of artesunate involves DNA damage mediated by reactive oxygen species. Antimicrob. Agents Chemother..

[39] Freese R. (2006). Markers of oxidative DNA damage in human interventions with fruit and berries. Nutr. Cancer.

[40] Uhlemann A.C., Wittlin S., Matile H., Bustamante L.Y., Krishna S. (2007). Mechanism of antimalarial action of the synthetic trioxolane RBX11160 (OZ277). Antimicrob. Agents Chemother..

[41] Postma N.S., Mommers E.C., Eling W.M.C., Zuidema J. (1996). Oxidative stress in malaria; implications for prevention and therapy. Pharm. World Sci..

[42] Percário S., Moreira D.R., Gomes B.A.Q., Ferreira M.E.S., Gonçalves A.C.M., Laurindo P.S.O.C., Vilhena T.C., Dolabela M.F., Green M.D. (2012). Oxidative stress in Malaria. Int. J. Mol. Sci..

[43] Tiwari S., Sharma N., Sharma G.P., Mishra N. (2021). Redox interactome in malaria parasite Plasmodium falciparum. Parasitol. Res..

[44] Loria P., Miller S., Foley M., Tilley L. (1999). Inhibition of the peroxidative degradation of haem as the basis of action of chloroquine and other quinoline antimalarials. Biochem. J..

[45] Zhang J., Krugliak M., Ginsburg H. (1999). The fate of ferriprotorphyrin IX in malaria infected erythrocytes in conjunction with the mode of action of antimalarial drugs. Mol. Biochem. Parasitol..

[46] Egan T.J., Combrinck J.M., Egan J., Hearne G.R., Marques H.M., Ntenteni S., Sewell B.T., Smith P.J., Taylor D., Van Schalkwyk D.A. (2002). Fate of haem iron in the malaria parasite Plasmodium falciparum. Biochem. J..

[47] Rahlfs S., Schirmer R.H., Becker K. (2002). The thioredoxin system of Plasmodium falciparum and other parasites. Cell. Mol. Life Sci..

[48] Desakorn V., Dondorp A.M., Silamut K., Pongtavornpinyo W., Sahassananda D., Chotivanich K., Pitisuttithum P., Smithyman A.M., Day N.P.J., White N.J. (2005). Stage-dependent production and release of histidine-rich protein 2 by Plasmodium falciparum. Trans. R. Soc. Trop. Med. Hyg..

[49] Wrenger C., Eschbach M.L., Müller I.B., Warnecke D., Walter R.D. (2005). Analysis of the vitamin B6 biosynthesis pathway in the human malaria parasite Plasmodium falciparum. J. Biol. Chem..

[50] Nickel C., Rahlfs S., Deponte M., Koncarevic S., Becker K. (2006). Thioredoxin networks in the malarial parasite Plasmodium falciparum. Antioxid. Redox Signal..

[51] Goldberg D.E., Sigala P.A. (2017). Plasmodium heme biosynthesis: To be or not to be essential?. PLOS Pathog..

[52] Müller S. (2004). Redox and antioxidant systems of the malaria parasite Plasmodium falciparum. Mol. Microbiol..

[53] Kawazu S., Komaki-Yasuda K., Oku H., Kano S. (2008). Peroxiredoxins in malaria parasites: Parasitologic aspects. Parasitol. Int..

[54] Mustacich D., Powis G. (2000). Thioredoxin reductase. Biochem. J..

[55] Slater A.F.G., Cerami A. (1992). Inhibition by chloroquine of a novel haem polymerase enzyme activity in malaria trophozoites. Nature.

[56] Gupta P., Mehrotra S., Sharma A., Chugh M., Pandey R., Kaushik A., Khurana S., Srivastava N., Srivastava T., Deshmukh A. (2017). Exploring Heme and Hemoglobin Binding Regions of Plasmodium Heme Detoxification Protein for New Antimalarial Discovery. J. Med. Chem..

[57] Choi C.Y.H., Cerda J.F., Hsiu-An C., Babcock G.T., Marletta M.A. (1999). Spectroscopic characterization of the heme-binding sites in Plasmodium falciparum histidine-rich protein 2. Biochemistry.

[58] Sullivan D.J., Gluzman I.Y., Goldberg D.E. (1996). Plasmodium Hemozoin Formation Mediated by Histidine-Rich Proteins. Science.

[59] Kapishnikov S., Grolimund D., Schneider G., Pereiro E., McNally J.G., Als-Nielsen J., Leiserowitz L. (2017). Unraveling heme detoxification in the malaria parasite by in situ correlative X-ray fluorescence microscopy and soft X-ray tomography. Sci. Rep..

[60] Sigala P.A., Goldberg D.E. (2014). The peculiarities and paradoxes of Plasmodium heme metabolism. Annu. Rev. Microbiol..

[61] Wallace W.J., Houtchens R.A., Maxwell J.C., Caughey W.S. (1982). Mechanism of autooxidation for hemoglobins and myoglobins. Promotion of superoxide production by protons and anions. J. Biol. Chem..

[62] Carrell R.W., Winterbourn C.C., Rachmilewitz E.A. (1975). Annotation: Activated oxygen and haemolysis. Br. J. Haematol..

[63] Thomas C., Mackey M., Diaz A., Cox D. (2009). Hydroxyl radical is produced via the Fenton reaction in submitochondrial particles under oxidative stress: Implications for diseases associated with iron accumulation. Redox Rep..

[64] Jani D., Nagarkatti R., Beatty W., Angel R., Slebodnick C., Andersen J., Kumar S., Rathore D. (2008). HDP—A novel heme detoxification protein from the malaria parasite. PLoS Pathog..

[65] Lew V.L., Tiffert T., Ginsburg H. (2003). Excess hemoglobin digestion and the osmotic stability of Plasmodium falciparum-Infected red blood cells. Blood.

[66] Mairbäurl H., Weber R.E. (2012). Oxygen transport by hemoglobin. Compr. Physiol..

[67] Clark I.A., Rockett K.A. (1996). Nitric oxide and parasitic disease. Adv. Parasitol..

[68] Ghigo D., Todde R., Ginsburg H., Costamagna C., Gautret P., Bussolino F., Ulliers D., Giribaldi G., Deharo E., Gabrielli G. (1995). Erythrocyte stages of Plasmodium falciparum exhibit a high nitric oxide synthase (NOS) activity and release an NOS-inducing soluble factor. J. Exp. Med..

[69] Egwu C.O., Pério P., Augereau J.-M., Tsamesidis I., Benoit-Vical F., Reybier K. (2021). Resistance to artemisinin in falciparum malaria parasites: A redox-mediated phenomenon. Free Radic. Biol. Med..

[70] Nogueira F., Diez A., Radfar A., Pérez-Benavente S., Rosario V.E., Puyet A., Bautista J.M. (2010). Early transcriptional response to chloroquine of the Plasmodium falciparum antioxidant defence in sensitive and resistant clones. Acta Trop..

[71] Wang W., Huang P., Jiang N., Lu H., Zhang D., Wang D., Zhang K., Wahlgren M., Chen Q. (2018). A thioredoxin homologous protein of Plasmodium falciparum participates in erythrocyte invasion. Infect. Immun..

[72] Fairfield A.S., Meshnick S.R., Eaton J.W. (1983). Malaria parasites adopt host cell superoxide dismutase. Science.

[73] Ginsburg H., Famin O., Zhang J., Krugliak M. (1998). Inhibition of glutathione-dependent degradation of heme by chloroquine and amodiaquine as a possible basis for their antimalarial mode of action. Biochem. Pharmacol..

[74] Porras P., Pedrajas J.R., Martínez-Galisteo E., Alicia Padilla C., Johansson C., Holmgren A., Antonio Bárcena J. (2002). Glutaredoxins catalyze the reduction of glutathione by dihydrolipoamide with high efficiency. Biochem. Biophys. Res. Commun..

[75] Patzewitz E.-M., Müller S. (2010). Glutathione biosynthesis and metabolism in Plasmodium falciparum. Malar. J..

[76] Lüersen K., Walter R.D., Müller S. (2000). Plasmodium falciparum-infected red blood cells depend on a functional glutathione de novo synthesis attributable to an enhanced loss of glutathione. Biochem. J..

[77] Patzewitz E.M., Wong E.H., Müller S. (2012). Dissecting the role of glutathione biosynthesis in Plasmodium falciparum. Mol. Microbiol..

[78] Harvey J.W., Beutler E. (1982). Binding of heme by glutathione S-transferase: A possible role of the erythrocyte enzyme. Blood.

[79] Müller S. (2015). Role and regulation of glutathione metabolism in Plasmodium falciparum. Molecules.

[80] Hirt R.P., Müller S., Martin Embley T., Coombs G.H. (2002). The diversity and evolution of thioredoxin reductase: New perspectives. Trends Parasitol..

[81] Bozdech Z., Ginsburg H. (2004). Antioxidant defense in Plasmodium falciparum - Data mining of the transcriptome. Malar. J..

[82] Lillig C.H., Holmgren A. (2007). Thioredoxin and related molecules - From biology to health and disease. Antioxid. Redox Signal..

[83] Yano K., Komaki-Yasuda K., Kobayashi T., Takemae H., Kita K., Kano S., Kawazu S.I. (2005). Expression of mRNAs and proteins for peroxiredoxins in Plasmodium falciparum erythrocytic stage. Parasitol. Int..

[84] Akerman S.E., Müller S. (2003). 2-Cys peroxiredoxin PfTrx-Px1 is involved in the antioxidant defence of Plasmodium falciparum. Mol. Biochem. Parasitol..

[85] Koncarevic S., Rohrbach P., Deponte M., Krohne G., Prieto J.H., Yates J., Rahlfs S., Becker K. (2009). The malarial parasite Plasmodium falciparum imports the human protein peroxiredoxin 2 for peroxide detoxification. Proc. Natl. Acad. Sci. USA.

[86] Atamna H., Pascarmona G., Ginsburg H. (1994). Hexose-monophosphate shunt activity in intact Plasmodium falciparum-infected erythrocytes and in free parasites. Mol. Biochem. Parasitol..

[87] Campanale N., Nickel C., Daubenberger C.A., Wehlan D.A., Gorman J.J., Klonis N., Becker K., Tilley L. (2003). Identification and characterization of heme-interacting proteins in the malaria parasite, Plasmodium falciparum. J. Biol. Chem..

[88] Ehrenshaft M., Bilski P., Li M., Chignell C.F., Daub M.E. (1999). A highly conserved sequence is a novel gene involved in de novo vitamin B6 biosynthesis. Proc. Natl. Acad. Sci. USA.

[89] Bilski P., Li M.Y., Ehrenshaft M., Daub M.E., Chignell C.F. (2000). Vitamin B6 (Pyridoxine) and Its Derivatives Are Efficient Singlet Oxygen Quenchers and Potential Fungal Antioxidants. Photochem. Photobiol..

[90] Matxain J.M., Padro D., Ristilä M., Strid Å., Eriksson L.A. (2009). Evidence of high ·OH radical quenching efficiency by vitamin B 6. J. Phys. Chem. B.

[91] Natera J., Massad W., García N.A. (2012). The role of vitamin B6 as an antioxidant in the presence of vitamin B2-photogenerated reactive oxygen species. A kinetic and mechanistic study. Photochem. Photobiol. Sci..

[92] Kannan K., Jain S.K. (2004). Effect of vitamin B 6 on oxygen radicals, mitochondrial membrane potential, and lipid peroxidation in H 2 O 2 -treated U937 monocytes. Free Radic. Biol. Med..

[93] Tsamesidis I., Egwu C.O., Pério P., Augereau J.M., Benoit-Vical F., Reybier K. (2020). An LC–MS assay to measure superoxide radicals and hydrogen peroxide in the blood system. Metabolites.

[94] Sullivan D.J., Matile H., Ridley R.G., Goldberg D.E. (1998). A common mechanism for blockade of heme polymerization by antimalarial quinolines. J. Biol. Chem..

[95] Sugioka Y., Suzuki M., Sugioka K., Nakano M. (1987). A ferriprotoporphyrin IX-chloroquine complex promotes membrane phospholipid peroxidation A possible mechanism for antimalarial action. FEBS Lett..

[96] Haynes R.K., Cheu K.W., Chan H.W., Wong H.N., Li K.Y., Tang M.M.K., Chen M.J., Guo Z.F., Guo Z.H., Sinniah K. (2012). Interactions between Artemisinins and other Antimalarial Drugs in Relation to the Cofactor Model-A Unifying Proposal for Drug Action. ChemMedChem.

[97] De Almeida Ribeiro M.C., Augusto O., Da Costa Ferreira A.M. (1995). Inhibitory effect of chloroquine on the peroxidase activity of ferriprotoporphyrin IX. J. Chem. Soc. Dalt. Trans..

[98] Birth D., Kao W.C., Hunte C. (2014). Structural analysis of atovaquone-inhibited cytochrome bc 1 complex reveals the molecular basis of antimalarial drug action. Nat. Commun..

[99] Fry M., Pudney M. (1992). Site of action of the antimalarial hydroxynaphthoquinone, 2-[trans-4-(4’-chlorophenyl) cyclohexyl]-3- hydroxy-1,4-naphthoquinone (566C80). Biochem. Pharmacol..

[100] Srivastava I.K., Rottenberg H., Vaidya A.B. (1997). Atovaquone, a broad spectrum antiparasitic drug, collapses mitochondrial membrane potential in a malarial parasite. J. Biol. Chem..

[101] Barton V., Fisher N., Biagini G.A., Ward S.A., O’Neill P.M. (2010). Inhibiting Plasmodium cytochrome bc1: A complex issue. Curr. Opin. Chem. Biol..

[102] Fiorillo M., Lamb R., Tanowitz H.B., Mutti L., Krstic-Demonacos M., Cappello A.R., Martinez-Outschoorn U.E., Sotgia F., Lisanti M.P. (2016). Repurposing atovaquone: Targeting mitochondrial complex III and OXPHOS to eradicate cancer stem cells. Oncotarget.

[103] Kevin Park B., O’Neill P.M., Maggs J.L., Pirmohamed M. (1998). Safety assessment of peroxide antimalarials: Clinical and chemical perspectives. Br. J. Clin. Pharmacol..

[104] Nosten F., White N.J. (2007). Artemisinin-based combination treatment of falciparum malaria. Am. J. Trop. Med. Hyg..

[105] Rudrapal M., Chetia D. (2016). Endoperoxide antimalarials: Development, structural diversity and pharmacodynamic aspects with reference to 1,2,4-trioxane-based structural scaffold. Drug Des. Devel. Ther..

[106] Posner G.H., Oh C.H. (1992). A Regiospecifically Oxygen-18 Labeled 1,2,4-Trioxane: A Simple Chemical Model System To Probe the Mechanism(s) for the Antimalarial Activity of Artemisinin (Qinghaosu). J. Am. Chem. Soc..

[107] Mercer A.E., Copple I.M., Maggs J.L., O’Neill P.M., Park B.K. (2011). The role of heme and the mitochondrion in the chemical and molecular mechanisms of mammalian cell death induced by the artemisinin antimalarials. J. Biol. Chem..

[108] Posner G.H., Oh C.H., Wang D., Gerena L., Milhous W.K., Meshnick S.R., Asawamahasadka W. (1994). Mechanism-Based Design, Synthesis, and in Vitro Antimalarial Testing of New 4-Methylated Trioxanes Structurally Related to Artemisinin: The Importance of a Carbon-Centered Radical for Antimalarial Activity. J. Med. Chem..

[109] Robert A., Benoit-Vical F., Claparols C., Meunier B. (2005). The antimalarial drug artemisinin alkylates heme in infected mice. Proc. Natl. Acad. Sci. USA.

[110] Zhang S., Gerhard G.S. (2008). Heme activates artemisinin more efficiently than hemin, inorganic iron, or hemoglobin. Bioorg. Med. Chem..

[111] Xie S.C., Dogovski C., Hanssen E., Chiu F., Yang T., Crespo M.P., Stafford C., Batinovic S., Teguh S., Charman S. (2016). Haemoglobin degradation underpins the sensitivity of early ring stage Plasmodium falciparum to artemisinins. J. Cell Sci..

[112] Wang J., Huang L., Li J., Fan Q., Long Y., Li Y., Zhou B. (2010). Artemisinin directly targets malarial mitochondria through its specific mitochondrial activation. PLoS ONE.

[113] Meunier B., de Visser S.P., Shaik S. (2004). Mechanism of oxidation reactions catalyzed by cytochrome P450 enzymes. Chem. Rev..

[114] Dawson J.H. (1988). Probing structure-function relations in heme-containing oxygenases and peroxidases. Science.

[115] Fita I., Rossmann M.G. (1985). The active center of catalase. J. Mol. Biol..

[116] Edwards S.L., Kraut J., Poulos T.L. (1988). Crystal Structure of Nitric Oxide Inhibited Cytochrome c Peroxidase. Biochemistry.

[117] Allen R.J.W., Kirk K. (2004). The Membrane Potential of the Intraerythrocytic Malaria Parasite Plasmodium falciparum. J. Biol. Chem..

[118] Biagini G.A., Viriyavejakul P., O’Neill P.M., Bray P.G., Ward S.A. (2006). Functional characterization and target validation of alternative complex I of Plasmodium falciparum mitochondria. Antimicrob. Agents Chemother..

[119] Antoine T., Fisher N., Amewu R., O’Neill P.M., Ward S.A., Biagini G.A. (2014). Rapid kill of malaria parasites by artemisinin and semi-synthetic endoperoxides involves ROS-dependent depolarization of the membrane potential. J. Antimicrob. Chemother..

[120] WHO Artemisinin Resistance and Artemisinin-Based Combination Therapy Efficacy (Status Report—August 2018). https://apps.who.int/iris/bitstream/handle/10665/274362/WHO-CDS-GMP-2018.18-eng.pdf?ua=1.

[121] Van Vugt M., Brockman A., Gemperli B., Luxemburger C., Gathmann I., Royce C., Slight T., Looareesuwan S., White N.J., Nosten F. (1998). Randomized comparison of artemether-benflumetol and artesunate-mefloquine in treatment of multidrug-resistant falciparum malaria. Antimicrob. Agents Chemother..

[122] Benoit-Vical F., Lelièvre J., Berry A., Deymier C., Dechy-Cabaret O., Cazelles J., Loup C., Robert A., Magnaval J.F., Meunier B. (2007). Trioxaquines are new antimalarial agents active on all erythrocytic forms, including gametocytes. Antimicrob. Agents Chemother..

[123] Leang R., Barrette A., Bouth D.M., Menard D., Abdur R., Duong S., Ringwald P. (2013). Efficacy of dihydroartemisinin-piperaquine for treatment of uncomplicated Plasmodium falciparum and Plasmodium vivax in Cambodia, 2008 to 2010. Antimicrob. Agents Chemother..

[124] Bopp S., Magistrado P., Wong W., Schaffner S.F., Mukherjee A., Lim P., Dhorda M., Amaratunga C., Woodrow C.J., Ashley E.A. (2018). Plasmepsin II-III copy number accounts for bimodal piperaquine resistance among Cambodian Plasmodium falciparum. Nat. Commun..

[125] Toler S. (2005). The plasmodial apicoplast was retained under evolutionary selective pressure to assuage blood stage oxidative stress. Med. Hypotheses.

[126] Storm J. (2012). Lipoic Acid Metabolism of Plasmodium—A Suitable Drug Target. Curr. Pharm. Des..

[127] Birnbaum J., Scharf S., Schmidt S., Jonscher E., Maria Hoeijmakers W.A., Flemming S., Toenhake C.G., Schmitt M., Sabitzki R., Bergmann B. (2020). A Kelch13-defined endocytosis pathway mediates artemisinin resistance in malaria parasites. Science.

[128] Klonis N., Crespo-Ortiz M.P., Bottova I., Abu-Bakar N., Kenny S., Rosenthal P.J., Tilley L. (2011). Artemisinin activity against Plasmodium falciparum requires hemoglobin uptake and digestion. Proc. Natl. Acad. Sci. USA.

[129] Ariey F., Witkowski B., Amaratunga C., Beghain J., Langlois A.C., Khim N., Kim S., Duru V., Bouchier C., Ma L. (2014). A molecular marker of artemisinin-resistant Plasmodium falciparum malaria. Nature.

[130] Witkowski B., Lelièvre J., Barragán M.J.L., Laurent V., Su X.Z., Berry A., Benoit-Vical F. (2010). Increased tolerance to artemisinin in Plasmodium falciparum is mediated by a quiescence mechanism. Antimicrob. Agents Chemother..

[131] Witkowski B., Khim N., Chim P., Kim S., Ke S., Kloeung N., Chy S., Duong S., Leang R., Ringwald P. (2013). Reduced artemisinin susceptibility of Plasmodium falciparum ring stages in western cambodia. Antimicrob. Agents Chemother..

[132] Peatey C., Chen N., Gresty K., Anderson K., Pickering P., Watts R., Gatton M.L., McCarthy J., Cheng Q. (2021). Dormant Plasmodium falciparum Parasites in Human Infections Following Artesunate Therapy. J. Infect. Dis..

[133] Clarebout G., Slomianny C., Delcourt P., Leu B., Masset A., Camus D., Dive D. (1998). Status of Plasmodium falciparum towards catalase. Br. J. Haematol..

[134] Varadharajan S., Sagar B.K.C., Rangarajan P.N., Padmanaban G. (2004). Localization of ferrochelatase in Plasmodium falciparum. Biochem. J..

[135] Bonday Z.Q., Dhanasekaran S., Rangarajan P.N., Padmanaban G. (2000). Import of host δ-aminolevulinate dehydratase into the malarial parasite: Identification of a new drug target. Nat. Med..

[136] Rocamora F., Zhu L., Liong K.Y., Dondorp A., Miotto O., Mok S., Bozdech Z. (2018). Oxidative stress and protein damage responses mediate artemisinin resistance in malaria parasites. PLoS Pathog..

[137] Mok S., Ashley E.A., Ferreira P.E., Zhu L., Lin Z., Yeo T., Chotivanich K., Imwong M., Pukrittayakamee S., Dhorda M. (2015). Population transcriptomics of human malaria parasites reveals the mechanism of artemisinin resistance. Science.

[138] Mbengue A., Bhattacharjee S., Pandharkar T., Liu H., Estiu G., Stahelin R.V., Rizk S.S., Njimoh D.L., Ryan Y., Chotivanich K. (2015). A molecular mechanism of artemisinin resistance in Plasmodium falciparum malaria. Nature.

[139] Tawk L., Chicanne G., Dubremetz J.F., Richard V., Payrastre B., Vial H.J., Roy C., Wengelnik K. (2010). Phosphatidylinositol 3-phosphate, an essential lipid in Plasmodium, localizes to the food vacuole membrane and the apicoplast. Eukaryot. Cell.

[140] Isozumi R., Uemura H., Kimata I., Ichinose Y., Logedi J., Omar A.H., Kaneko A. (2015). Novel mutations in k13 propeller gene of artemisinin-resistant Plasmodium falciparum. Emerg. Infect. Dis..

[141] Balikagala B., Mita T., Ikeda M., Sakurai M., Yatsushiro S., Takahashi N., Tachibana S.I., Auma M., Ntege E.H., Ito D. (2017). Absence of in vivo selection for K13 mutations after artemether-lumefantrine treatment in Uganda. Malar. J..

[142] Lee A.H., Fidock D.A. (2016). Evidence of a mild mutator phenotype in cambodian Plasmodium falciparum malaria parasites. PLoS ONE.

[143] Paloque L., Witkowski B., Lelièvre J., Ouji M., Ben Haddou T., Ariey F., Robert A., Augereau J.M., Ménard D., Meunier B. (2018). Endoperoxide-based compounds: Cross-resistance with artemisinins and selection of a Plasmodium falciparum lineage with a K13 non-synonymous polymorphism. J. Antimicrob. Chemother..

[144] Schirmer R.H., Coulibaly B., Stich A., Scheiwein M., Merkle H., Eubel J., Becker K., Becher H., Müller O., Zich T. (2003). Methylene blue as an antimalarial agent. Redox Rep..

[145] McNamara C.W., Lee M.C.S., Lim C.S., Lim S.H., Roland J., Nagle A., Simon O., Yeung B.K.S., Chatterjee A.K., McCormack S.L. (2013). Targeting Plasmodium PI(4)K to eliminate malaria. Nature.

[146] Tiwari N.K., Reynolds P.J., Calderón A.I. (2016). Preliminary LC-MS Based Screening for Inhibitors of Plasmodium falciparum Thioredoxin Reductase (PfTrxR) among a Set of Antimalarials from the Malaria Box. Molecules.

[147] Vallières C., Avery S.V. (2017). The candidate antimalarial drug mmv665909 causes oxygen-dependent mRNA mistranslation and synergizes with quinoline-derived antimalarials. Antimicrob. Agents Chemother..

